# Progress in Basic Research and Clinical Strategies for Cementum Regeneration

**DOI:** 10.1016/j.identj.2025.02.017

**Published:** 2025-03-24

**Authors:** Xiaoxue Zhu, Dandan Xiang, Yiding Huo, Xiaotao He, Faming Chen, Beimin Tian, Xuan Li

**Affiliations:** State Key Laboratory of Oral & Maxillofacial Reconstruction and Regeneration, National Clinical Research Center for Oral Diseases, Shaanxi International Joint Research Center for Oral Diseases, Department of Periodontology, School of Stomatology, The Fourth Military Medical University, Xi'an, China

**Keywords:** Cementum regeneration, Periodontitis, Periodontal regeneration, Growth factors, Stem cell-based therapy

## Abstract

Periodontitis is a chronic inflammatory disease that inflicts damage to periodontal tissues, leading to loss of teeth, and affects systemic health. Traditional treatments can delay inflammation, whereas regeneration of the periodontal complex (periodontal ligament, cementum, and alveolar bone) can better restore periodontal tissue function. In recent years, the regeneration of alveolar bone and the periodontal ligament has been widely reviewed although cementum has received less attention. As an avascular mineralised structure around the tooth, cementum can anchor periodontal ligament fibres to the root surface, thereby connecting teeth to alveolar bone. The supporting and stabilizing effects of cementum make its regeneration vital for restoring the functionality of the periodontal tissues. In this review, we discuss advancements in basic and clinical research appertaining to cementum regeneration. We describe the molecular mechanisms that contribute to cementum regeneration thereby providing a foundation for further basic research. Finally we summarise the clinical strategies employed for cementum regeneration, including regenerative surgery and utilisation of growth factors and stem cells.

## Introduction

Periodontitis is a common oral disease that irreversibly damages periodontal tissue, including the periodontal ligament (PDL), cementum, and alveolar bone, which support dentition. This disease affects 90% of people worldwide and leads to inflammatory disorders of the periodontal tissue and even other organs.^1^ On the basis of findings from the Global Burden of Disease Study 2019, there has been a global escalation in the age-standardised rates of periodontitis prevalence, incidence, and disability-adjusted life years from 1990 to 2019.^2^ Consequently, researchers are dedicated to studying treatments for periodontal disease.

Over the past few decades, several traditional treatments, such as scaling and root planning have been commonly used and have achieved significant clinical effects. Such treatments delay the process of inflammation by removing plaque and calculus but are unable to restore the original connective tissue and reconstruct the intricate organisation of the periodontal complex (PDL, cementum, and alveolar bone).^3^ Thus, functional restoration of the multitissue periodontal complex is needed. In this process, one of the key steps is to attach PDL to the mineralisation layer formed on the surface of the bone and tooth root.^4^ Some regeneration surgeries, such as guided tissue regeneration (GTR) and bone grafting, are effective only for bone regeneration but have yielded limited success in regenerating the structural and functional aspects of cementum, which reminds us to pay more attention to cementum regeneration.

The cementum is an avascular mineralisation structure that connects teeth to alveolar bone through PDL fibres.^5^ The most important function of the cementum is to serve as an anchor point for the main PDL fibres on the surface of the tooth root. Such anchoring helps maintain the occlusal relationship and supports the teeth through a markedly dynamic response.^6^ The regeneration of cementum is considered essential for fostering the attachment of new fibrous tissues, maintaining the integrity of the PDL, and preventing the occurrence of tooth ankylosis.^4^ The stability of teeth and their protection against resorption depend heavily on the integrity of the cementum. Periodontal inflammation damages the cells and the cementum on the tooth root, leading to the growth of the epithelium and the formation of a crevice where bacteria accumulate. The loss of cementum disrupts the microbial barrier and leads to the deterioration of periodontitis.^7^ Research indicates that individuals with cementum deficiency or abnormalities are prone to early-onset periodontitis, which results in abnormal attachment and increases the vulnerability of teeth to periodontal pathogens.^8^ Therefore, the regeneration of cementum, especially when it incorporates functional fibres, is vital for the development of periodontal tissue and is an important step in periodontal regeneration.^4^^,^^9^

However, owing to the complex structure of cementum, the understanding of how it regenerates still lags well behind the knowledge regarding other periodontal tissue.^6^^,^^10^ The cementogenic procedure for PDL attachments remains a challenge in periodontal regeneration.^11^ Considering that cementum generation is important for its support, protection, and fixation effects on teeth and is crucial for preserving the PDL and regenerating periodontal tissue, we summarise the current basic research and clinical technologies related to cementum regeneration. It is anticipated that periodontal tissue regenerative medicine will ultimately benefit the clinical treatment of periodontitis.

## Classification and development of cementum

### Classification of cementum

**Fig. 1:**
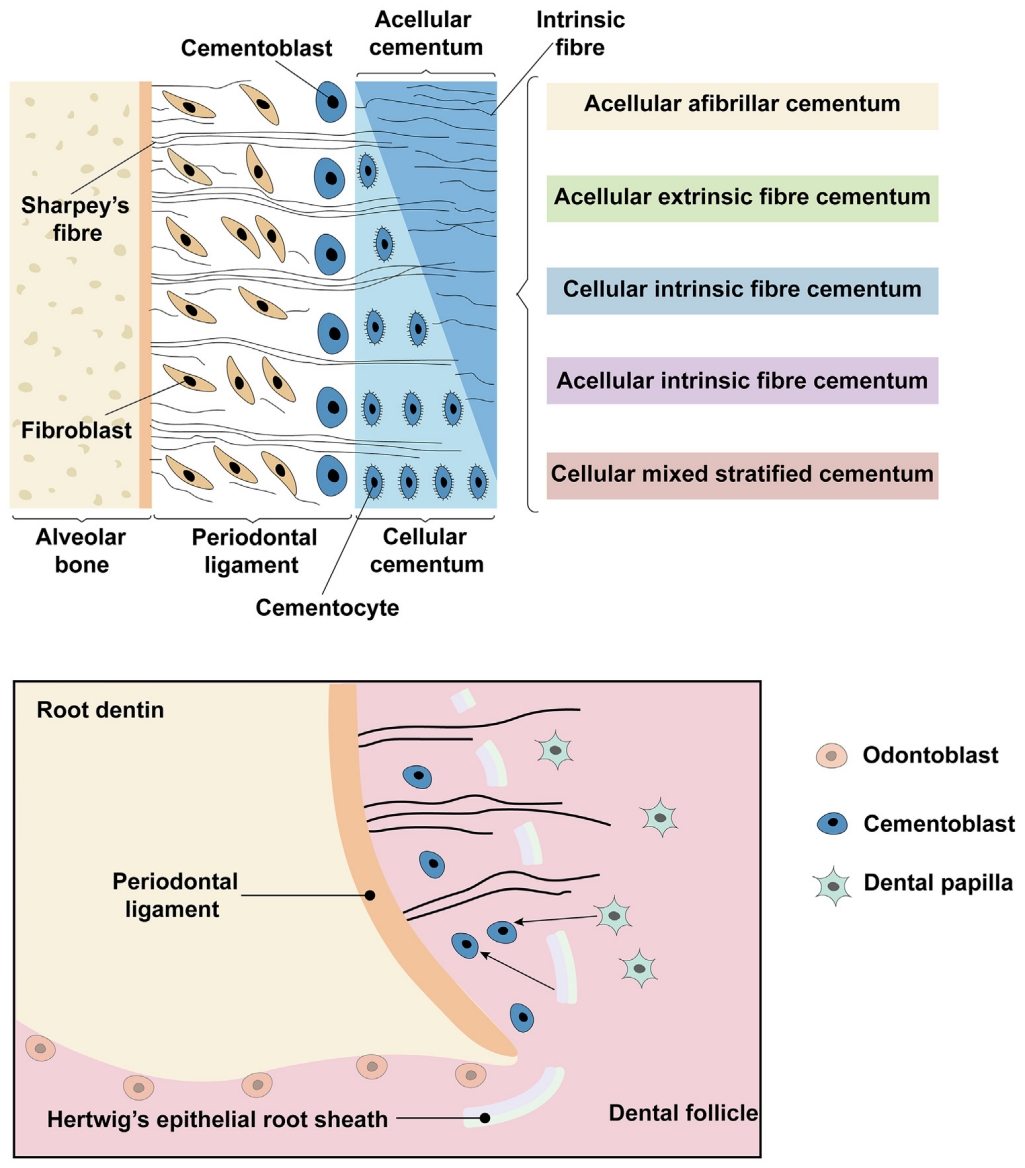
Classification and development of cementum. Schematic diagram of the classification of cementum, including acellular afibrillar cementum (AAC), acellular extrinsic fibre cementum (AEFC), cellular intrinsic fibre cementum (CIFC), acellular intrinsic fibre cementum (AIFC) and cellular mixed stratified cementum (CMSC). Dental follicle cells (DFCs) and parts of Hertwig's epithelial root sheath (HERS) differentiate into cementoblasts and subsequently form cementum matrix.

Histologically, cementum can be categorised into cellular cementum and acellular cementum on the basis of whether it contains cementocytes.^12^ There are 2 varieties of collagen fibres in the cementum. Extrinsic fibres, namely, perforating fibres or Sharpey's fibres, are partly generated by PDL fibroblasts and partly by cementoblasts, serving a crucial function in anchoring the cementum to the alveolar bone. Thus, the formation of Sharpey's fibres facilitates recovery of the functional regeneration of cementum. Intrinsic fibres are inherent fibres created by cementoblasts.^13^ On the basis of the distribution of the cementocytes and the origin of the collagen fibres, cementum can be classified into 5 detailed categories: acellular afibrillar cementum (AAC), acellular extrinsic fibre cementum (AEFC), cellular intrinsic fibre cementum (CIFC), acellular intrinsic fibre cementum (AIFC) and cellular mixed stratified cementum (CMSC) (Figure 1). Among these types, AEFC and CMSC are embedded with abundant Sharpey's fibres,^5^^,^^14^ which play essential roles in cementum regeneration. The AEFC is located at the cervical root surface. It contains no cells and is composed of densely arranged collagen fibre bundles (also referred to as Sharpey's fibres, which are perpendicular to the root surface). CMSC is composed of a mixture of multiple areas of AEFC and CIFC that cover the interradicular and apical areas of roots.^13^^,^^14^ In summary, differentiation into various types of cementum is highly important for its generation.

### Development of cementum

The supporting periodontal tissue (including the cementum, alveolar bone, and PDL) all develop from dental follicles.^15^ The ectomesenchymal cells from the dental follicle differentiate into cementoblasts and ultimately form cementum.

During dental tissue development, the inner and outer enamel epithelium at the cervical loop extends from the apex and forms Hertwig's epithelial root sheath (HERS). The HERS can enhance the differentiation and immigration of dental follicle cells (DFCs) through appropriate stimulation, which is important for root development.^16^ The dental papilla cells enveloped by HERS also proliferate toward the apex and are induced to become odontoblasts, thereby forming root dentin. The HERS fractures after root dentin formation, allowing contact between the DFCs and the root dentin. DFCs subsequently differentiate into cementoblasts. Some portions of the HERS also differentiate into cementoblasts^16^^,^^17^ and ultimately form a cementum matrix, into which collagen fibres are secreted and simultaneously embedded.^18^

## Molecular mechanisms related to cementum regeneration

**Fig. 2:**
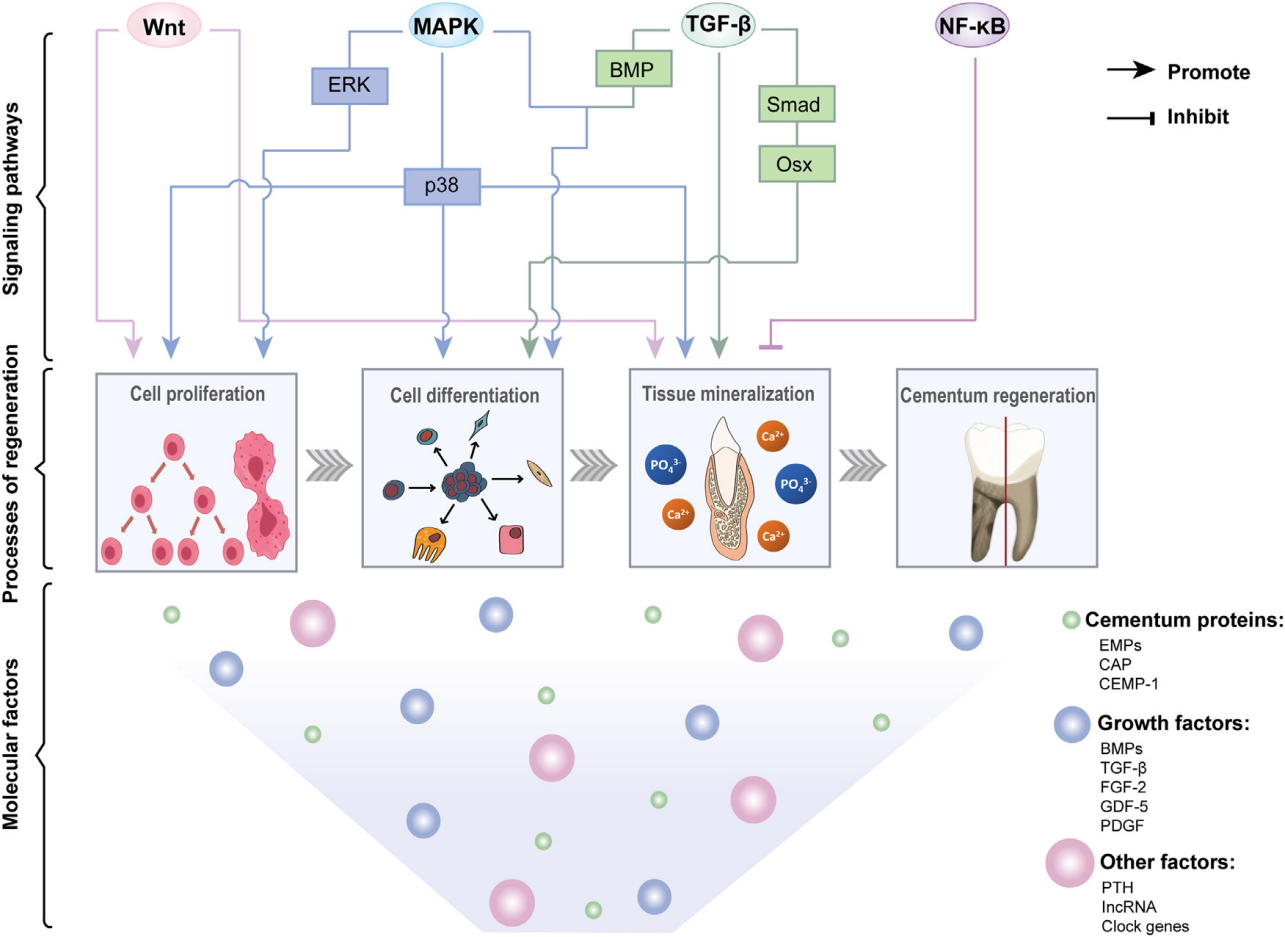
Molecular mechanisms related to cementum regeneration. The molecular factors that contribute to cementum regeneration can be divided into 2 categories, namely, cementum proteins and growth factors. The cementum proteins include mainly cementum protein-1 (CEMP-1), cementum attachment protein (CAP) and enamel matrix proteins (EMPs). Among the related growth factors, bone morphogenetic proteins (BMPs), transforming growth factor-β (TGF-β), fibroblast growth factor-2 (FGF-2), growth/differentiation factor-5 (GDF-5), and platelet-derived growth factor (PDGF) are considered critical. In addition, other factors, such as parathyroid hormone (PTH), long noncoding RNAs (lncRNAs) and clock genes, play key roles in related biochemical processes. These factors activate specific signalling pathways, mainly the Wnt, TGF-β, mitogen-activated protein kinase (MAPK), and nuclear factor kappa-B (NF-κB) signalling pathways, promoting the regeneration of cementum by stimulating cell differentiation and proliferation and enhancing mineralisation.

For a deeper comprehension of cementum regeneration, it is essential to understand the composition of and molecular factors associated with cementum. The main organic components of the cementum are collagen proteins, with type I collagen being the predominant form, accounting for 90% of the total collagen proteins in the cementum.^19^ Its main function is to maintain the integrity of the cementum structure. Noncollagenous proteins, including bone sialoprotein (BSP), cementum attachment protein (CAP), and cementum protein-1 (CEMP-1), have their respective functions in the cementum. The strategy of cementum regeneration typically involves the intricate interplay of cementum-related proteins, growth factors, and diverse signalling pathways, accompanied by cell proliferation, differentiation, and tissue mineralisation, ultimately leading to the regeneration of cementum (Figure 2).

### Cementum proteins

The cementum proteins mainly include CEMP-1, CAP, and enamel matrix proteins (EMPs). They can induce signalling pathways related to mitogenesis, increase the Ca^2+^ concentration, initiate the protein kinase C cascade, and enhance the migration and adhesion of progenitor cells.^7^ The above mechanism leads to the differentiation of cementoblasts and the production of cementum-like mineralised tissue.

### CEMP-1

CEMP-1 is a protein expressed in cementoblasts and their progenitor cells that functions to enhance mineralisation, proliferation, differentiation, and cell maturation.^7^ CEMP-1 overexpression can enhance the proliferation and differentiation of PDL cells, thereby inducing a mineralizing phenotype, improving alkaline phosphatase (ALP) activity, and producing a cementum-like mineralised matrix, which can be applied for periodontal regeneration.^7^^,^^20^ For example, transfecting CEMP-1 into human gingival fibroblasts (HGFs) can induce mineralisation and the expression of cementum proteins.^21^ A previous study revealed that recombinant human CEMP-1 (rhCEMP-1) can affect the nucleation and growth of hydroxyapatite crystals, promoting the formation of cementum.^22^ Another study reported that synthetic CEMP-1-derived peptide (CEMP-1-p1) can facilitate the formation of CMSC, AEFC, and PDL and the embedding of newly formed Sharpey's fibres.^23^ The relevant mechanism may be that hCEMP-1 can upregulate cementogenic genes such as run-related transcription factor 2 (RUNX2), bone morphogenetic protein-2 (BMP-2), secreted phosphoprotein 1 and CAP, thereby promoting cementum formation.^24^ In summary, CEMP-1 is a specific protein with multiple characteristics that can promote cell mineralisation, differentiation, and maturation and induce cementum formation. Synthetic CEMP-1-p1 has been utilised in cementogenic research, which provides possibilities for periodontal regeneration.

### CAP

CAP is a specific protein located in the cementum that plays a regulatory role in cementum formation and serves as a biomarker for evaluating the situation of cementum regeneration.^25^^,^^26^ CAP can promote the adhesion and differentiation of DFCs, recruit cementoblastic progenitors and enhance their differentiation.^27^^,^^28^ Furthermore, CAP has the capacity to stimulate the formation of cementum-like tissue from periodontal cells.^29^ Human recombinant calcium attachment protein (hrPTPLA/CAP), a CAP derivative prepared with CAP-specific antibodies, has been proven to promote hydroxyapatite crystal formation and new bone generation.^27^ Peptides derived from CAP can promote the development of carbonate hydroxyapatite crystals and the proliferation and migration of human PDL cells.^30^ Multiple experiments have confirmed that EMPs, BMP-2, BMP-7, CEMP-1, etc., are capable of stimulating the differentiation of cementoblasts and enhancing cementum regeneration by increasing the expression of CAP.^21^^,^^31^^,^^32^ Therefore, CAP has great potential for tissue mineralisation and cementum regeneration.

### EMPs

EMPs are a group of proteins primarily composed of amelogenin (AM) that promote cementum regeneration. The component purified from developing porcine tooth enamel is called the enamel matrix derivative (EMD), and owing to its unique characteristics, most research has focused on the application of cementum regeneration.^33^ EMD effectively repairs periodontal defects by promoting the mineralisation of human DFCs, allowing their differentiation into cementoblasts.^34^ EMD can also promote PDL cells to proliferate, differentiate, and produce extracellular matrix (ECM),^7^ which may induce AEFC or CIFC to form.^35^ Calcitriol has a mineralizing effect and can induce gene expression for cementum formation. Compared with calcitriol, EMD enhances the expression of CEMP-1 and CAP to promote cementum induction of human PDL-derived cells in a later stage with less mineralisation.^36^ The major component of EMD, AM, promotes cementoblasts proliferation and differentiation, and its C-terminus promotes the cementogenic differentiation of human cementoblast lineage cells by increasing the expression of ALP, osteocalcin (OCN), and BSP.^37^

### Growth factors

#### BMPs

BMPs, which belong to the transforming growth factor-β (TGF-β) superfamily, are abundantly expressed in mineralised tissues, and participate in the process of bone and cartilage formation.^38^ Many studies have revealed that BMPs can promote the differentiation of DFCs into osteoblasts and cementoblasts; thus, BMPs are considered important factors in promoting periodontal regeneration.^39^ BMP-2 induces DFCs to differentiate toward a cementoblast phenotype in a manner that is dependent on both the dosage and duration of exposure. Moreover, BMP-2 promotes the expression of BSP, OCN, etc., and enhances mineralisation.^40^^,^^41^ In contrast, BMP-3 may inhibit these processes. Studies have shown that BMP-3 can counteract the osteoblastic differentiation induced by BMP-2, and its inhibitory impact on mineralisation makes it possible to maintain the PDL between bone and cementum.^42^ More basic and clinical studies have shown that BMP-2 promotes periodontal complex regeneration.^43^^,^^44^ In combination with dentin sialoprotein (DSP), recombinant human BMP-2 (rhBMP-2) can stimulate the growth and differentiation of cementoblasts via autocrine BMP, integrin, and the Wnt/β-catenin, mitogen-activated protein kinase (MAPK), and nuclear factor kappa-B (NF-κB) pathways.^45^

Furthermore, BMP-7 exhibits significant promise across numerous facets of dental tissue regeneration and is specifically localised to the cementum.^46^^,^^47^ BMP-7 promotes cementum regeneration by inducing osteoblastic/cementoblastic differentiation of periodontal ligament stem cells (PDLSCs) and dental follicle progenitor cells, increasing the expression of the RUNX2 and OCN mRNAs during this process. This process also depends on the dose and time. The relevant mechanism may involve the induction of procollagen C-proteinase enhancer 1 (PCPE1) and BMP-­1 by BMP-7. These factors play crucial roles in the maturation of type I collagen, facilitating the differentiation and mineralisation processes within cementoblasts.^48^ BMP-7 regulates the high expression of protein tyrosine phosphatase-like member A/CAP (PTPLA/CAP) in human PDL cells, via the involvement of GC-rich Smad-binding elements (GC-SBEs).^49^ Compared with BMP-2, BMP-7 also facilitates the integration of cementum on the dentin surface.^47^

#### TGF-β

TGF-β can regulate the transcription factor Osterix (Osx), which is involved in cellular cementum formation through the Smad transmission pathway, thereby promoting cementoblast differentiation and cementum formation.^50^ TGF-β1 is present in the periodontal complex and participates in periodontal regeneration. TGF-β1 does not affect cell proliferation but promotes the differentiation and mineralisation of cementoblasts. Studies have revealed that ALP, BSP and type I collagen mRNA expression are increased by TGF-β1, which promotes the differentiation and mineralisation of cementoblasts and the development of cementum.^51^ As mentioned above, fragmentation of the HERS is essential for the formation of cementum, and the HERS can differentiate into cementoblasts, which can potentially enable cementum regeneration. TGF-β1 can regulate HERS fragmentation via epithelial‒mesenchymal transition (EMT), and this fragmentation can promote cementum formation.^52^^,^^53^ In addition, recombinant human transforming growth factor β3 promotes the expression of TGF-β3, CEMP-1, and OCN, while regulating BMP-2 and osteogenic protein-1 (OP-1) to induce cementum regeneration.^54^

#### Fibroblast growth factor-2 (FGF-2)

Experimental evidence from various animal studies indicates that FGF-2 and growth/differentiation factor-5 (GDF-5) contribute significantly to cementum and bone formation.^55^ The FGF family is instrumental in the germination of teeth and the development of mineralised tissue.^56^ As HERS can differentiate into cementoblasts themselves, FGF-2 can accelerate this process via EMT.^52^ Many animal studies and clinical trials have demonstrated the efficacy of FGF-2 in regenerating periodontal tissue.^57^, ^58^, ^59^, ^60^ FGF promotes the formation of new bone, cementum, and PDL by accelerating the proliferation of fibroblasts and enhancing angiogenesis.

#### Growth Differntiation Factor -5 (GDF-5)

GDF-5 is another member of the TGF-β/BMP family that regulates cell differentiation and bone formation.^61^ GDF-5 stimulates human PDL cell proliferation.^62^ As induced pluripotent stem cells (iPSCs) have been utilised to regenerate periodontal tissue, GDF-5 can induce cementum regeneration in iPSCs by increasing the mRNA expression levels of key markers (such as CAP and CEMP-1) associated with the cementum.^63^

#### Platelet-derived growth factor (PDGF)

PDGF acts as a regulatory molecule that can stimulate cell chemotaxis, proliferation, and matrix synthesis. PDGF is capable of swiftly recruiting PDL cells. When used in conjunction with other growth factors, such as BMP-7 and insulin-like growth factor 1 (IGF-1), it can facilitate the effective regeneration of cementum and periodontal tissue.^64^^,^^65^ IGF-1 stimulates the proliferation and differentiation of human PDLSCs into PDL fibroblasts, whereas PDGF promotes regenerative cementum mainly through its chemotactic and mitogenic effects on PDLSCs.^66^

More factors can affect cementum regeneration. Parathyroid hormone (PTH) maintains calcium balance and affects cementum formation. Intermittent delivery of PTH induces the expression of cementoblastic proteins and promotes cementum regeneration of cementoblasts through cyclic AMP-dependent protein kinase A (PKA), extracellular signal-regulated kinases 1/2 (ERK1/2), and the Wnt pathway.^67^^,^^68^ Long noncoding RNAs (lncRNAs) such as the Lgr6 intergenic transcript under intermittent PTH (LITTIP) and GACAT2, play roles in cementum regeneration,^67^^,^^69^ with H19 (a long noncoding RNA) assisting in differentiation and mineralisation.^70^ In addition, LINC01444 mediates the regulation of cementoblastic differentiation by melatonin in an inflammatory environment. The inhibition of LINC01444 by melatonin can promote the cementoblastic differentiation of PDLSCs.^71^ Clock genes in cementum facilitate regeneration by stimulating cell growth and mineralisation.^72^ Bmal1, a key clock gene, enhances cementum formation and mineralisation by increasing the expression of specific markers and promotes mineralised nodule formation through the Wnt/β-catenin pathway.^73^ REV-ERBs, circadian proteins, also affect cementoblast functions and cellular cementum formation by controlling the expression of Osx, which influences cell proliferation and mineralisation.^74^ Moreover, the mineralisation and differentiation of cementoblasts are also impacted by factors secreted by macrophages. The M1 polarisation markers interleukin (IL)-1β, IL-6, and tumor necrosis factor-α (TNF-α) were found to weaken the mineralisation of cementoblasts, whereas the M2 polarisation marker peroxisome proliferator activated receptor γ (PPARγ) stimulates cementoblast mineralisation.^75^ The macrophages can polarise toward the M2 phenotype affected by PDLSCs,^76^ which then promotes the differentiation of PDLSCs into cementoblasts by activating the Akt and c-Jun N-terminal kinase (JNK) signalling pathways.^77^

### Relevant signalling pathways

#### Wnt/β-catenin signalling pathway

The Wnt signalling pathway, particularly the Wnt/β-catenin pathway, plays crucial roles in various stages of tissue repair and regeneration, including cementum regeneration. Both in vitro and in vivo experiments have demonstrated that activation of Wnt can promote the formation of cementum. The activation of the Wnt signalling pathway can notably enhance the mineralisation process, increase ALP activity, and upregulate the expression of genes and proteins associated with bone and cementum, including OCN, osteopontin (OPN), CEMP-1, and CAP, thus inducing cementum deposition and the formation of PDL fibres.^31^ Activation of Wnt/β-catenin signal transduction in epithelial cells can promote tooth development.^78^ As the basis of cementum, the function of cementoblasts is regulated by a variety of cytokines and signal transduction pathways. Studies have shown that the Wnt/β-catenin signalling pathway is closely related to the growth of cementoblasts, and that specifically, Wnt1 can promote the proliferation of cementoblasts without affecting their differentiation.^79^ In addition, Li et al.^67^ reported that PTH promotes cementum regeneration because the LITTIP/ Lgr6/ heterogeneous nuclear ribonucleoprotein K (HnRNPK) complex can regulate the production of cementum through Wnt signalling.

#### TGF-β signalling pathway

The TGF-β superfamily includes BMP, GDF, activin, and TGF-β. In the cementum, TGF-β was significantly expressed in cementoblasts. Previous studies have reported that TGF-β signal transduction regulates Osx expression through a Smad-dependent pathway, thereby affecting cementoblast differentiation and cementum formation.^80^ Moreover, Smads and activating protein-1 (AP-1) activation of TGF-β signalling can upregulate the transcription of osteophorin in cementoblasts to inhibit osteoclast generation, thereby protecting cementum from absorption.^81^ Unlike the Wnt/β-catenin signalling pathway, TGF-β1 does not affect human cementoblast (HCEM) proliferation but promotes cell differentiation and mineralisation processes.^51^ Moreover, the BMP pathway can promote cell growth and cementoblastic differentiation. For example, recombinant human acoustic hedgehog (rh-SHH) activates downstream Smad1/5/8 by promoting the expression of the BMP-2/BMP-4 genes.^82^ Furthermore, with the participation of the MAPK signalling pathway, BMP-2 can induce dental capsule cells to differentiate into cementoblasts in a time- and dose-dependent manner.^41^

### MAPK signalling pathway

The MAPK cascade is a pivotal element within a network of vital signalling pathways. This family encompasses several key subfamilies, such as the ERK (extracellular-signal-regulated kinase), JNK, and p38/SAPK (stress-activated protein kinase) subfamilies, which play significant roles in the process of cementum formation.^83^ In a study of *Porphyromonas gingivalis (P.gingivalis)*, researchers reported that *P.gingivalis* can inhibit cementoblast differentiation and mineralisation by regulating CXXC‐type zinc finger protein 5 (CXXC5), which involves mainly the MAPK, phosphoinositide 3-kinase (PI3K)-Akt and Wnt signalling pathways.^84^ In addition, the autophagy and apoptosis of cementoblasts can also be activated through the Stat3/ERK/Akt signalling cascade.^85^ Extracts of EMPs used in periodontal regeneration, such as AM, promote the proliferation of HCEMs via the MAPK‒ERK signalling pathway.^86^ Another study on irisin revealed that it enhances the proliferation, differentiation and mineralisation process of cementoblasts by increasing p38 MAPK signal transduction.^87^

### NF-κB signalling pathway

NF-κB is a nuclear transcription factor that has been shown to negatively regulate mineralisation in osteoblasts and other cells. Yes-associated proteins have been shown to inhibit NF-κB pathway activity and promote cementum formation.^88^ Deficiency of the transcription factor 7-like 2 (Tcf7l2) in the transcription factor E protein family can inhibit NF-κB activation and cementum formation, providing guidance for cementum regeneration.^89^

There are more signalling pathways involved in the generation of cementum, and notably, these signalling pathways do not function independently. The inhibition of differentiation and induction of apoptosis in cementoblasts by TNF-α mainly relies on the activity of p53. The p38, ERK 1/2, JNK, PI3K-Akt, and NF-κB pathways are also activated.^90^ MiR-361-3p inhibits cementoblast differentiation and mineralisation by targeting nuclear factor of activated T-cell 5 (Nfat5), which involves the ERK1/2 and PI3K-Akt pathways. The JNK, p38, NF-κB, and Wnt/β-catenin pathways also modulate this axis.^91^ Furthermore, various signalling pathways have their own limitations and effects. For example, while the Wnt and TGF-β signalling pathways are intricately involved in the regulation of cell behaviour during cementum regeneration, the Wnt signalling pathway seems to have more controversial effects, with both promoting and inhibiting effects on cementum regeneration reported. Current data from investigations of the role of canonical Wnt signalling in cementum formation, however, are controversial and inconsistent. Cementum formation can be inhibited when the Wnt/β-catenin pathway is activated or potentially overactivated.^92^ The proliferation and differentiation of cementoblasts may differ in terms of the intensity of the Wnt signalling pathway.^93^ Therefore, accurately regulating the Wnt signalling pathway to regenerate the cementum is a key issue. While the TGF-β signalling pathway is essential for cementum regeneration and plays a crucial role in various cellular processes, its effects can be context dependent and influenced by the surrounding microenvironment.^80^ Understanding these limitations and optimizing TGF-β signalling could enhance therapeutic strategies for periodontal regeneration.

These signalling pathways have shown certain potential in cementum regeneration through various molecules. However, many unknown factors and limitations remain, and further research is needed to elucidate how to precisely regulate these signalling pathways to achieve optimal therapeutic effects.

From the discussion of the above molecules, we can conclude that cementoblasts are the key cells involved in cementum regeneration. We can measure the levels of markers associated with cementum, including CEMP-1, CAP, ALP, OCN, and mineralised nodules, to evaluate the regenerative effect. Some biological factors that function through various signalling pathways are relatively mature in the field of periodontal regeneration and have shown good therapeutic effects in clinical trials (Table 1). However, the specific mechanisms of many applications still need to be explored.

**Table 1:** Molecular mechanisms related to cementum regeneration.

Molecular factors	Growth factor/Protein/Pathway	Functional effects in cementum regeneration	References
Cementum proteins	CEMP-1	Induces a mineralizing phenotype, improves ALP activity, produces a cementum-like mineralised matrix, and stimulates the proliferation and migration of PDL cells	^7^
	CAP	Promotes the adhesion and differentiation of DFCs, recruits cementoblastic progenitors and enhances their differentiation, stimulates the formation of cementum-like tissue. and regulates cell recruitment and differentiation	^7^^,^^27^
	EMPs	Promote the mineralisation and differentiation of DFCs, promote the proliferation and differentiation of PDL cells and the production of ECM, and enhances CEMP-1 and CAP expression	^7^^,^^34^^,^^36^
Growth factors	BMPs	Induce DFCs to differentiate into a cementoblast phenotype, stimulate the growth and differentiation of cementoblast, and induce cementoblast differentiation of PDLSCs and dental follicle progenitor cells	^41^^,^^45^^,^^48^
	TGF-β	Regulates Osx expression, promotes differentiation and mineralisation of cementoblasts, and regulates HERS fragmentation	^50^^,^^51^^,^^52^
	FGF-2	Accelerates the differentiation of HERS into cementoblasts and the proliferation of fibroblasts	^52^^,^^57^
	GDF-5	Stimulates human PDL cells proliferation and increases the mRNA expression levels of CAP and CEMP-1	^62^^,^^63^
	PDGF	Stimulates cell chemotaxis, proliferation, and matrix synthesis and recruits PDL cells	^65^
Signalling pathways	Wnt/β-catenin	Enhances the mineralisation, boosts ALP activity, and upregulates the expression of genes and proteins associated with bone and cementum	^31^
	TGF-β	Affects cementoblast differentiation and promotes cell mineralisation	^82^
	MAPK	Enhances the proliferation, differentiation and mineralisation process of cementoblasts	^87^
	NF-κB	Regulates cell mineralisation negatively	^88^

## Clinical strategies for cementum regeneration

**Fig. 3:**
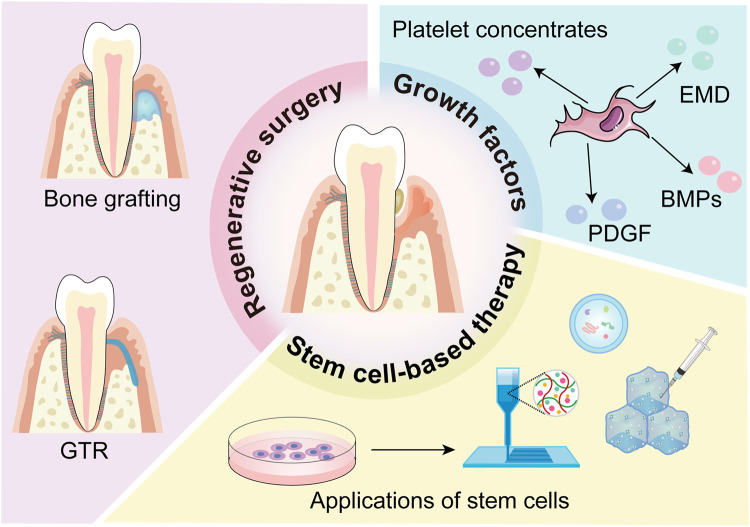
Schematic representation of the clinical techniques used in periodontal regeneration therapy, including regenerative surgery, the application of growth factors, and stem cell-based therapy.

The commonly used techniques for periodontal regeneration in clinical applications include regenerative surgery, the utilisation of growth factors, and stem cell-based therapy (Figure 3). These regenerative methods, whether applied individually or in combination, greatly increase the clinical effectiveness of periodontal regeneration, but they also have shortcomings and limitations. Above, we described the important role of cementum regeneration in periodontal regeneration. Here, we concentrate on the impact of various clinical methods on cementum regeneration.

### Regenerative surgery

Regenerative surgery is designed to promote the regeneration of periodontal attachment structures, that is, to form new cementum on the infected tooth root and functional PDL and to reattach the teeth to the newly regenerated alveolar bone.

### Bone grafting

Bone grafting involves implanting bone or bone substitutes into alveolar bone defects caused by periodontitis to promote periodontal regeneration. The commonly used transplant materials for bone grafting include autologous bone, allogeneic bone, xenogeneic bone, and alloplastic substitutes. The selection of materials substantially influences the effectiveness of tissue regeneration. According to a previous study, for corresponding intrabony defects, the formation of new cementum measured 1.9 mm for autologous bone grafts, 1.3 mm for allogeneic bone, 2.4 mm for xenogeneic bone, and 0.6 mm for alloplastic substitutes calculated linearly along the tooth surface.^94^ Autologous bone, as the most biocompatible graft biomaterial, can achieve greater regenerative efficacy when combined with EMD.^95^ Controlled trials have demonstrated that the combination of platelet-rich plasma (PRP), autologous bone, and GTR has better therapeutic effects on cementum formation than does GTR alone, with autologous bone playing a major role.^96^

### GTR

GTR refers to the use of membranous materials as barriers in periodontal surgery to block the growth of the gingival epithelium along the root surface during healing; this provides a certain space and enables PDL cells with regenerative ability to preferentially occupy the root surface, forming new attachments and promoting periodontal regeneration.^97^ Nine studies providing data on the impact of GTR on intrabony defects reported a mean formation of 2.6 mm cementum.^94^ Regarding the efficacy of GTR, the selection of barrier membranes is a key factor that includes 2 categories: resorbable (such as collagen) and nonresorbable (such as polytetrafluoroethylene and Ti).^98^

However, the curative effect of GTR alone is limited and unpredictable. For example, membrane exposure leading to microbial contamination could result in unsuccessful outcomes in regenerative surgery.^99^ The combination of barrier membranes and other biomolecules and technologies often results in better efficacy in cementum regeneration; however, these methods still have not achieved long-term and stable clinical effects.^100^

### The application of growth factors

Growth factors facilitate intercellular communication by binding to specific receptors on target cells, thereby regulating cell proliferation, differentiation, and apoptosis.^101^ Currently, the growth factors closely related to cementum regeneration include FGF-2, TGF-β, EMD, PDGF, GDF-5, and BMP. These growth factors are commonly used in surgeries such as GTR, bone grafting, and tissue engineering to promote the postoperative effect of periodontal surgery. The FDA has approved several growth factors, including EMD, PDGF, rhBMP-2, and PRP, for use in periodontal regeneration, and their curative effects have also been confirmed.^102^

One of the most commonly used growth factors in the clinic is EMD. The clinical effects of EMD have also been confirmed through various experimental models, which have shown periodontal regeneration with newly formed bone, cementum, and inserted fibres.^103^^,^^104^ EMD combined with an autogenous bone graft can stimulate the regeneration of periodontal tissue, including cementum, Sharpey's fibres, and PDL, indicating its high effectiveness as a therapeutic.^105^ As stem cell-based therapy and other cutting-edge therapies have advanced, EMD combined with various materials has been increasingly researched for regeneration.^106^ Furthermore, 3 types of autologous platelet concentrates are commonly employed in clinical practice for periodontal regeneration: PRP, platelet-rich fibrin (PRF) and concentrated growth factor (CGF). Specifically, PRP is used for hard and soft tissue surgery; PRF is used to treat gingival recession, furcation, and intrabony defects; and CGF is used for bone regeneration.^107^ Compared with PRP, PRF and CGF contain more growth factors.^108^ Several animal experiments have confirmed that the use of PRP can promote the formation of cementum-like tissue.^109^, ^110^, ^111^ Additionally, the application of PRF to intrabony defects in beagle dogs results in the regeneration of cementum; in particular, the use of PRF produced via horizontal centrifugation is the best therapy.^112^ Injectable PRF (i-PRF) is an advanced form of PRF.^113^ A meta-analysis revealed that i-PRF can greatly improve periodontal parameters and has a notable effect on clinical therapy.^114^^,^^115^ To date, there have been few clinical studies on the use of autologous platelet concentrates in the field of cementum regeneration, and this topic deserves further attention.

Additionally, the combination of rhBMP-2 and an absorbable collagen sponge (ACS) carrier can effectively induce cementum formation in clinical studies.^116^ Furthermore, rhBMP-2-pretreated hPDLSCs promoted regeneration of the mineralised layer with embedded PDLs.^9^ Current studies are concentrating on the therapeutic impact of growth factors in clinical applications, with case studies and expert analyses offering significant insights. Biological agents such as recombinant human platelet-derived growth factor-BB (rhPDGF-BB) and recombinant human fibroblast growth factor-2 (rhFGF-2) have been deemed safe and efficacious, exhibiting histological indications of new bone, cementum, and PDL formation.^117^

### Stem cell-based therapy

#### Sources of stem cells

The stem cells used in periodontal regeneration can be divided into dental-derived stem cells and non-dental-derived stem cells. The dental-derived stem cells mainly include PDLSCs, stem cells from human exfoliated deciduous teeth (SHEDs), stem cells from the apical papilla (SCAPs), and dental follicle stem cells (DFSCs), which have the potential for cementum regeneration. These cells function mainly by further differentiating into cementoblasts and regenerating cementum-like tissue, PDL fibres, and periodontal vascular regeneration in vivo.^118^ PDLSCs have great potential for periodontal regeneration.^119^ A series of animal experiments and clinical trials have shown that PDLSCs and DFSCs can promote cementum regeneration, generating a cementum-like structure with inserted collagen fibres.^118^^,^^120^ PDLSCs can also differentiate into osteogenic and fibrogenic lineages, with greater potential to form the periodontal complex.^121^ Some studies have demonstrated the effectiveness and clinical safety of PDLSCs therapy in regenerating the PDL, but the practical application of this method has not yet been determined.^122^

Moreover, some non-dental stem cells commonly used in clinical research, such as bone marrow-derived mesenchymal stem cells (BMMSCs), adipose-derived stem cells (ADSCs), and iPSCs, also promote cementum regeneration. Li *et al*.^123^ evaluated the regenerative efficacy of 5 common stem cells, including PDLSCs, BMMSCs, ADSCs, DPSCs, and gingiva-derived MSCs (GMSCs), in periodontal defect animal models. Among these cells, PDLSCs and BMMSCs demonstrate great regenerative potential, whereas ADSCs perform better in terms of cementum regeneration. In many animal models, ADSC implantation promotes regeneration of the cementum and PDL. Adding other scaffold materials or fibrin sealants can also improve regenerative conditions.^124^

#### Application forms of stem cells

The methods for using stem cells in periodontal regeneration include cell injection, cell sheets, and their combination with biomaterials. However, advanced applications, such as exosomes and clinical drugs derived from stem cells, have gradually attracted increasing attention as well.

*Cell injection:* As a minimally invasive technique, cell injection can be performed directly to prevent the trauma caused by surgery, but the effectiveness of cell injection is limited, and it is difficult to track, requiring cell labelling and the combination of additional techniques for improved outcomes. Shi *et al*.^125^ conducted research on periodontal regeneration by labelling PDLSCs with superparamagnetic iron oxide (SPIO) nanoparticles (PC-SPIO) and injecting them. An animal experiment examined the impact of injecting SCAPs in a minipig with periodontitis. The findings indicated that the regenerative cementum in the SCAP-injected group was thicker, wider, and more mature than that in the control group, and clinical indicators including probing depth, attachment level, and gingival recession, were significantly improved. However, further studies are needed to elucidate the detailed mechanisms involved.^126^

*Cell sheets:* Cell sheet technology capitalises on the innate capacity of cells to produce ECM on their own, which can simulate the cellular microenvironment and maintain the endogenous ECM to support cell growth; however, these sheets lack mechanical strength.^127^ Cell sheets made from PDL cells, BMMSCs, and alveolar periosteal cells can form a thin layer of mineralised cementum tissue on the surface of the tooth root and express CEMP-1 markers, which effectively meet the requirements of cementum regeneration. In addition, combining cell sheets with PDL and bone scaffolds can promote regeneration of the periodontal complex. Autologous PDL cell sheets that can be used for periodontal regeneration have passed clinical trials.^127^ Iwata *et al*.^128^ used 3-layered PDL cell sheets and porous β-tricalcium phosphate (β-TCP) to induce the formation of bone and cementum with well-oriented collagen fibres. In an animal study, rADSC cell sheets modified with the hCEMP-1 gene were used to repair periodontal defects in osteoporotic rats and achieved good results in cementum regeneration.^24^

*Combination with biomaterials:* The principle of tissue engineering regeneration involves the combination of 3 major elements, namely, biomaterial scaffolds, regenerative cells, and bioactive factors, which are used to construct biomimetic systems that induce new tissue formation. Scaffolds are practical biomaterials that maintain space, provide stability for blood clots, and promote the regeneration process in surgical wounds.^129^ This approach is designed to emulate the structure and function of the ECM, providing a new approach for regenerating defective tissues. The scaffold can maintain mechanical integrity, provide growth factors, and promote cell growth and differentiation. There are 3 main types of scaffold materials (including natural polymers, synthetic polymers, and bioceramics), which provide a 3-dimensional (3D) environment to organise regenerative cells.

Natural polymers, such as chitosan (CS), sodium alginate, hyaluronic acid (HA), and collagen, are directly sourced from natural resources. Natural biomaterials are biocompatible and biodegradable and can promote cellular reactions and wound healing.^130^ Yang *et al*.^131^ used bioskiving to prepare alternating collagen lamellae mimicking cementum, biomineralised them in fluorine-containing calcium phosphate, and successfully constructed biomimetic cementum. They further verified that this biomimetic cementum can induce true cementum formation. The application of cross-linked HA (xHyA) strongly promotes new cementum formation when fibres are inserted in class III furcation defects.^132^ A 3D-printed membrane composed of gelatin, elastin, and sodium hyaluronate has shown safety and efficacy in GTR for periodontal regeneration therapies, promoting cementum formation after 8 weeks in an animal study.^98^

Synthetic polymers are synthesised through chemical reactions, and common synthetic polymers include polyethylene glycol (PEG) and polylactic-co-glycolic acid (PLGA). Delivery of IGF-1 and BMP-6 via a CS/alginate/PLGA hybrid scaffold can induce the proliferation and differentiation of cementoblasts.^133^ Polycaprolactone (PCL) is also a common polymer that meets the needs of regenerative purposes in GTR. PCL-based scaffolds promote the regeneration of bone and cementum.^134^ Hydrogels, which are functional polymer materials composed mainly of natural and synthetic polymers, are also commonly used materials for periodontal regeneration. A scaffold composed of a hydrogel, other active molecules and materials can successfully promote the formation of the periodontal complex.^135^^,^^136^

Many bioceramics, such as hydroxyapatite (HA), TCP, and bioactive glass, target the regeneration of alveolar bone and cementum because of their high degradability. HA bioceramics with micro-nano-hybrid surface (mnHAs) can promote the expression of cementogenic-related markers and thus seem to be promising biomaterials for periodontal regeneration.^137^ Silicate-based bioceramics perform even better in terms of cementum formation.^138^ Some biomaterials, such as mineral trioxide aggregate (MTA) with β-TCP covered with a collagen membrane, are more effective at promoting cementum regeneration.^139^

The porosity of a material determines the exchange and communication of molecules and cells. In addition, nanomaterials can promote adhesion between cells and biomaterials.^102^ A new nanocomposite, nano-CaF_2_, can release Ca and F ions, therefore enhancing the cementogenic induction of PDLSCs.^140^ Some biphasic and triphasic scaffolds have been designed for periodontal regeneration and appear to be effective in cementum regeneration; the insertion of Sharpey's fibres and a cementum-like structure is observed.^141^^,^^142^ The key aspects of designing multiphase scaffolds for periodontal tissue engineering include compartmentalizing the bone and PDL, promoting the formation of cementum on the root surface and forming PDL fibres with appropriate orientation.^143^ The biphasic scaffold made by Yu *et al*.^144^ is similar to natural periodontal tissue, which successfully achieves regeneration with PDL fibres inserted into the newly formed cementum and alveolar bone. Layered scaffolds are designed for the regeneration of each layer of the periodontal complex. A 3-layer nanocomposite hydrogel scaffold tailored with different materials and growth factors for each layer of the periodontal complex has shown promising regenerative outcomes.^136^

3D printing techniques may also be employed in this area, as they allow precise control of the scaffold microstructure. The Incorporation of 3D-printed gelatin methacryloyl scaffolds with bioactive glass has shown the capacity to stimulate cementogenic activity and exhibit significant regenerative potential.^145^ 3D-printed periodontal patches yielded promising therapeutic outcomes in the functional regeneration of cementum and even the entire periodontal complex.^146^ Ono *et al*. employed 3D printing technology to create structures resembling PDL tissue and integrated these with bioactive materials, which led to the enhancement of gene expression associated with cementum and the formation of cementum-like structures.^147^

*Exosomes, Exos:* Exosomes are membrane vesicles that are secreted by stem cells and have characteristics similar to those of their parental cells. Exosomes are regarded as a natural nanoparticle delivery approach to cure disease because of their stability and high abundance of bioactive molecules; however, they are unsatisfactory in terms of loading and release. Currently, exosomes combined with various biomaterials have been used to attenuate inflammation and promote regeneration.^148^ The scaffolds used to load the exosomes for periodontal regeneration include hydrogels, collagen sponges, and β-TCP scaffolds, providing the exosomes with effective protection and fixation so that the exosomes can be better delivered and released. Deng *et al*.^149^ used a biotin-avidin system to deliver exosomes, thus enhancing their therapeutic performance and accelerating the regeneration of periodontal tissue including cementum, and the released hydrogel promoted M2 polarisation.

M1-polarised macrophages can weaken the mineralisation of cementoblasts, whereas M2-polarised macrophages enhance this process, in which exosomes play an important role.^75^ Because knocking out casein kinase 2 interacting protein-1 (Ckip-1) can promote macrophage polarisation toward the M2 phenotype, studies have shown that exosomes derived from Ckip-1-silenced macrophages can also improve the inhibitory effect of *P. gingivalis* on cementoblast mineralisation and cementum regeneration.^150^ By reducing severe endoplasmic reticulum stress and the unfolded protein response, exosomes from M2 macrophages containing melatonin have been shown to rejuvenate the ability of PDLCs to differentiate into cementoblasts.^151^

Sun *et al*.^152^ also reported that exosomes derived from human GMSCs diminish the inflammatory response of PDLSCs by modulating the NF-κB and Wnt5a signalling pathways, which is beneficial for regenerative therapy. Exosomes from human PDLSCs can promote the migration, proliferation, and mineralisation of cementoblasts through the PI3K/AKT signalling pathway.^153^ Currently, the utilisation of exosomes for cementum regeneration remains an underexplored area of research. While the number of targeted investigations into the use of exosomes for cementum regeneration is limited, their demonstrated anti-inflammatory effects, capacity to increase cell proliferation, and osteogenic differentiation suggest that they possess significant potential in this area and merit further exploration. In clinical applications, the production and modification of exosomes are also issues that need to be considered.^154^ It is crucial in regenerative medicine to assess the comparative roles of exosomes derived from various cell types in the induction of cementum regeneration.^148^

**Fig. 4:**
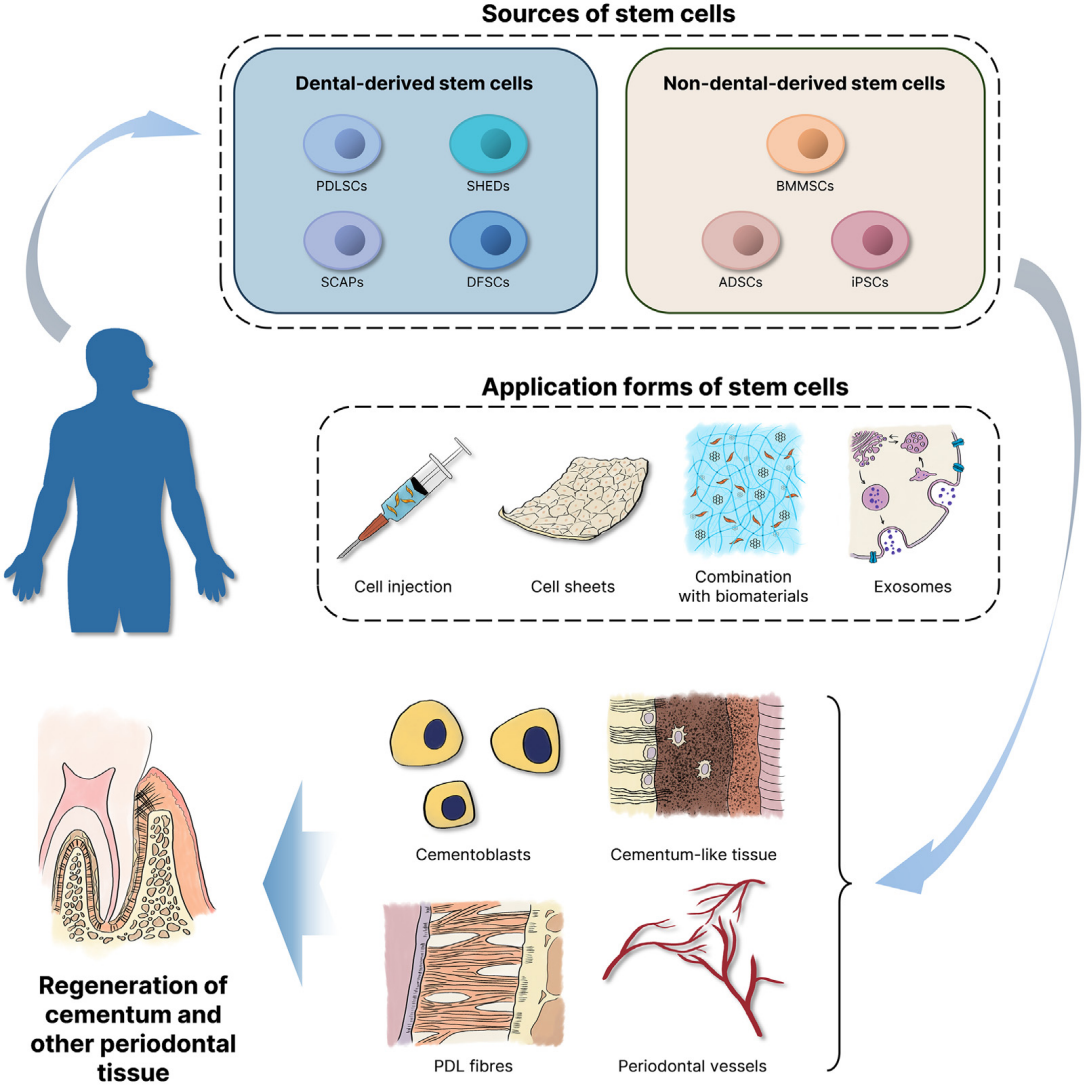
Application of stem cell-based therapy in cementum regeneration. Dental stem cells and non-dental stem cells can exert their effects through cell injection, cell sheets, binding with biomaterials, exosomes, and other methods. They can differentiate into cementoblasts, forming regenerated cementum-like tissue, periodontal ligament (PDL) fibres, and periodontal vessels.

In conclusion, there is widespread application of stem cell-based therapy in the field of periodontal regeneration. Both dental stem cells and non-dental stem cells have been proven to have good effects. The techniques, including cell injection, cell sheets, extensive combination with biomaterials, and the development of exosomes, are diverse and have shown certain therapeutic performance in the regeneration of cementum and other periodontal tissue (Table 2) (Figure 4). Furthermore, some new regeneration technologies are gradually emerging. For example, the development of stem cell-based clinical drugs is a focal point in periodontal regeneration. DPSCs, as promising choices for MSC drugs, are being investigated clinically for periodontal disease treatment.^160^ It is believed that in the future, there will be a greater investment in research on stem cell-based clinical drugs to achieve true transformation of clinical results. The application of MSC/ECM complexes induces the formation of reconstructed tissue, including cementum, in rat lesion areas, demonstrating a scaffold-free strategy in tissue engineering.^161^ In summary, attention should be given to the clinical translation of periodontal regeneration, especially for cementum.

**Table 2:** The advantages and disadvantages of various clinical strategies.

Clinical strategies	Specific methods	Advantages	Disadvantages	References
Regenerative surgery	Bone grafting (Take autogenous bone graft as an example)	Histocompatible, structural support, and has no risk of disease transmission	Multiple complications, limited supply	^94^^,^^155^
	GTR	Provides a physical barrier and avoids connective and epithelial tissue growing down into the defect	Limited and unpredictable effects, membrane exposure leading to microbial contamination, additional surgery for non-resorbable membranes' removal	^94^^,^^97^^,^^99^
The application of growth factors	Growth factors	Safe and effective, predictability	The effect is not significant when used alone, lack of tissue specificity	^156^^,^^117^
Stem cell-based therapy	Cell injection	Simple, minimally invasive and repeatable	Limited effectiveness, difficult to track in vivo, insufficient cell supply	^106^^,^^157^
	Cell sheets	Easier to deliver and transplant	Lack mechanical strength, high cost and a thickness limit in creating tissues	^107^^,^^108^^,^^158^
	Combination with biomaterials	Maintain mechanical integrity, provide growth factors	Inflammation caused by biodegradation of scaffolds, safety problems	^100^
	Exosomes	Stability and richness in bioactive molecules; low immunogenicity, high drug loading capacity, biocompatibility, specificity and stability, and lack of cytotoxicity	Rapid clearance by host cells, a short half-life, unsatisfactory in terms of loading and releasing	^148^^,^^159^

## Future challenges

The process of cementum regeneration is extremely complex, especially the reorganisation of oriented PDL fibres firmly attached to new cementum, as it requires precise control of cell differentiation and tissue integration. Translating basic research results into clinical applications is challenging.^118^ The problems encountered with the use of these technologies in clinical settings are equally deserving of our attention.

A range of growth factors have demonstrated therapeutic benefits in clinical periodontal regeneration. However, the utilisation of growth factors such as BMPs depends on supraphysiological concentrations, which not only increase expenses but also frequently result in complications, hindering the optimal clinical outcomes of BMPs. To increase the regenerative potency of growth factors and bolster their future clinical use, it is imperative to focus on the dosage, duration, and timing of growth factor administration to cells.^162^ Subsequent studies should concentrate on novel delivery systems and tailored scaffolds to facilitate the efficient and prolonged release of growth factors.^156^

While stem cell-based therapy offers considerable potential, various challenges must be addressed to move from experimental studies to practical clinical use. For example, the risk of tumour formation associated with undifferentiated human iPSCs is a significant concern. In addition, the lack of large-scale culture systems suitable for clinical applications, complex harvesting procedures, limited transplantation and survival, and cell aging may lead to practical difficulties in translating basic research into clinical applications.^163^ More advanced biomaterials and scaffolds can mimic the natural ECM and provide conducive cell growth and differentiation environments. The development of these materials must consider factors such as biocompatibility, biodegradability, and the ability to support the complex architecture of the periodontal tissue.^143^

Importantly, certain considerations that arise during the clinical implementation of these technologies should be considered. When growth factors are applied in clinical settings, it is essential to assess various factors related to the patient, their dental health, and the surgical procedure used to predict the success of regenerative treatments. Factors such as a patient's oral hygiene, smoking behavior, and extent of periodontal damage significantly impact the success of regenerative therapies, indicating the need for personalised treatment for patients. Maintaining optimal oral hygiene is vital for patients to achieve enduring success in periodontal regeneration. The integration of minimally invasive approaches with growth factors and bone grafts has yielded positive outcomes in clinical practice. A more in-depth understanding of these factors and the strategic use of growth factors can increase the likelihood of successful periodontal regeneration.^117^ In addition, periodontal defects are frequently associated with an inflammatory microenvironment. Inflammation can significantly impede the regenerative process, as it can lead to tissue destruction and impair the normal function of stem cells and other regenerative components. This necessitates a heightened demand for anti-inflammatory and immune regulatory capabilities in periodontal regeneration strategies.^164^

As tissue engineering has advanced, the exploration of various biomaterials has increased. While conventional materials boast the advantages of low cost and excellent biocompatibility, they have multiple complications. Innovative alternatives such as nanomaterials have demonstrated significant promise in the field of periodontal regeneration. However, their biological safety and biodegradability remain critical factors that must be carefully evaluated for their use in clinical periodontal regeneration.^165^

In summary, future research should focus on devising more potent systems for the delivery of growth factors, enhancing the biocompatibility and safety of regenerative materials. Tailored regeneration protocols must be crafted, considering the specific periodontal status of each patient. Furthermore, additional clinical trials are essential to ascertain the practicality of these technological implementations. By addressing these challenges and limitations, we can refine the clinical utilisation of cementum regeneration techniques, thereby optimizing treatment efficacy for patients.

## Conclusion

**Fig. 5:**
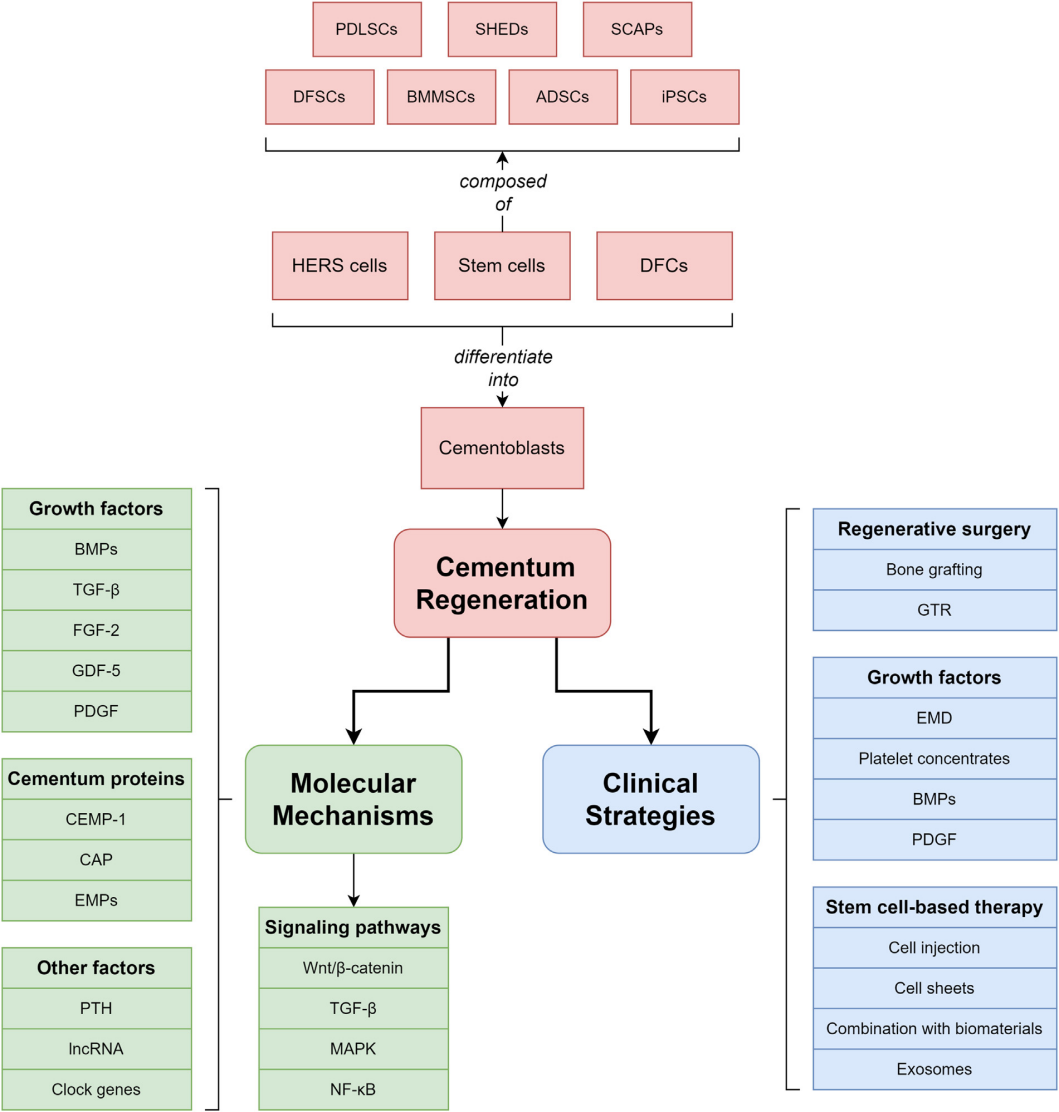
The key steps in cementum regeneration. Cementoblasts, as the key cells for cementum regeneration, can be differentiated from stem cells, Hertwig's epithelial root sheath (HERS) cells, and dental follicle cells (DFCs). The related molecular mechanisms include the involvement of growth factors, cementum proteins, and signalling pathways. Common clinical strategies include regenerative surgery, the application of growth factors, and stem cell-based therapy.

The reconstruction of periodontal attachment structures is the key to periodontal medicine. Research on periodontal regeneration should focus on promoting the formation of cementum on the root surface, periodontal fibre formation and embedment. This review discusses the progress in basic research and clinical practice related to cementum regeneration, including important molecules and mechanisms, as well as current beneficial methods in clinical practice, which all play important roles in supporting teeth and periodontal tissue. In current basic research and clinical practice, growth factors, stem cells, and biochemical materials have received much attention and have been studied for the treatment and regeneration of periodontal disease. In addition, increasingly advanced technologies such as exosome therapy, stem cell drugs, and novel materials are being applied in cementum regeneration (Figure 5).

Periodontal regeneration is a complex process involving multiple factors, and many issues related to the study of cementum regeneration remain. As previously discussed, the development of AEFC and CMSC is ideal for cementum regeneration, as they provide desirable attachment functions and high mineralisation. However, in the majority of periodontal regeneration studies, the cementum that forms is CIFC, which has a weaker attachment function, or the type of cementum regeneration is not a priority. Although growth factors offer potential for tissue regeneration, their broad range of activity results in a lack of tissue specificity. Additional studies are needed to determine the optimal timing and concentration of various growth factor deliveries, as well as to increase the compatibility among different growth factors to maximise regenerative outcomes. In addition, owing to the challenges encountered in the clinical application of stem cell transplantation, interest in endogenous regenerative approaches such as stem cell homing, which offer novel insights for periodontal regeneration, has increased.

Research will continue to be needed to restore the interfaces between soft and hard tissues, including the cementum-ligament-bone interface. Tissue engineering continues to be a focal point of research in periodontal regeneration, including cementum, with ongoing and future explorations likely to encompass areas such as endogenous regeneration, the development of innovative scaffold materials, the controlled delivery of growth factors, and advancements in drugs and gene therapies. It is crucial to focus on the particular regeneration of cementum, with the specific types of cementum being regenerated and maintaining the integrity of the entire periodontal complex. As recent advancements in research and technology have been promising, the introduction of various growth factors, biomaterials, and stem cell treatments has shown potential for clinical application in cementum regeneration. However, there remain significant challenges in translating these technologies into widespread clinical practice.

In summary, research on cementum regeneration is still limited, as cementum is often used as an additional observational indicator, and most studies still focus on the regeneration of the PDL and alveolar bone. With an increasing focus on the integral concept of the periodontal complex, there is a need to refine existing studies and subsequently validate them through animal models and human clinical trials to achieve the desired regenerative efficacy.

## Conflict of interest

None disclosed.

## References

[1] Pihlstrom B.L., Michalowicz B.S., Johnson NW. (2005). Periodontal diseases. Lancet.

[2] Wu L., Zhang S.Q., Zhao L., Ren Z.H., Hu CY. (2022). Global, regional, and national burden of periodontitis from 1990 to 2019: Results from the Global Burden of Disease study 2019. J Periodontol.

[3] Xu X.Y., Li X., Wang J., He X.T., Sun H.H., Chen FM. (2019). Concise review: periodontal tissue regeneration using stem cells: strategies and translational considerations. Stem Cells Transl Med.

[4] Park C.H., Oh J.H., Jung H.M. (2017). Effects of the incorporation of ε-aminocaproic acid/chitosan particles to fibrin on cementoblast differentiation and cementum regeneration. Acta Biomater.

[5] Nanci A., Bosshardt DD. (2006). Structure of periodontal tissues in health and disease. Periodontol 2000.

[6] Bosshardt D.D., Selvig KA. (1997). Dental cementum: the dynamic tissue covering of the root. Periodontol 2000.

[7] Arzate H., Zeichner-David M., Mercado-Celis G. (2015). Cementum proteins: role in cementogenesis, biomineralization, periodontium formation and regeneration. Periodontol 2000.

[8] Page R.C., Baab DA. (1985). A new look at the etiology and pathogenesis of early-onset periodontitis. Cementopathia revisited. J Periodontol.

[9] Park J.Y., Park C.H., Yi T., Kim S.N., Iwata T., Yun JH. (2020). rhBMP-2 Pre-treated human periodontal ligament stem cell sheets regenerate a mineralized layer mimicking dental cementum. Int J Mol Sci.

[10] Saygin N.E., Giannobile W.V., Somerman MJ. (2000). Molecular and cell biology of cementum. Periodontol.

[11] Saito M.M., Onuma K., Yamakoshi Y. (2023). Cementum is key to periodontal tissue regeneration: a review on apatite microstructures for creation of novel cementum-based dental implants. Genesis.

[12] Foster BL. (2017). On the discovery of cementum. J Periodontal Res.

[13] Yamamoto T., Hasegawa T., Yamamoto T., Hongo H., Amizuka N. (2016). Histology of human cementum: Its structure, function, and development. Jpn Dent Sci Rev.

[14] Schroeder HE. (1992). Biological problems of regenerative cementogenesis: synthesis and attachment of collagenous matrices on growing and established root surfaces. Int Rev Cytol.

[15] Zhou T., Pan J., Wu P. (2019). Dental follicle cells: roles in development and beyond. Stem Cells Int.

[16] Guo Y., Guo W., Chen J., Chen G., Tian W., Bai D. (2018). Are Hertwig's epithelial root sheath cells necessary for periodontal formation by dental follicle cells?. Arch Oral Biol.

[17] Huang X., Bringas P., Slavkin H.C., Chai Y. (2009). Fate of HERS during tooth root development. Dev Biol.

[18] Huang X.F., Chai Y. (2012). Molecular regulatory mechanism of tooth root development. Int J Oral Sci.

[19] Landis W.J., Jacquet R. (2013). Association of calcium and phosphate ions with collagen in the mineralization of vertebrate tissues. Calcif Tissue Int.

[20] Komaki M., Iwasaki K., Arzate H., Narayanan A.S., Izumi Y., Morita I. (2012). Cementum protein 1 (CEMP1) induces a cementoblastic phenotype and reduces osteoblastic differentiation in periodontal ligament cells. J Cell Physiol.

[21] Carmona-Rodríguez B., Alvarez-Pérez M.A., Narayanan A.S. (2007). Human cementum protein 1 induces expression of bone and cementum proteins by human gingival fibroblasts. Biochem Biophys Res Commun.

[22] Chen X., Liu Y., Yang J., Wu W., Miao L., Yu Y. (2016). The synthesis of hydroxyapatite with different crystallinities by controlling the concentration of recombinant CEMP1 for biological application. Mater Sci Eng C Mater Biol Appl.

[23] Hoz L., López S., Zeichner-David M., Arzate H. (2021). Regeneration of rat periodontium by cementum protein 1-derived peptide. J Periodontal Res.

[24] Wang M., He M., Xu X., Wu Z., Tao J., Yin F. (2023). Cementum protein 1 gene-modified adipose-derived mesenchymal stem cell sheets enhance periodontal regeneration in osteoporosis rat. J Periodontal Res.

[25] Yasunaga M., Ishikawa H., Tamaoki S., Maeda H., Ohno J. (2022). Embedded human periodontal ligament stem cells spheroids enhance cementogenic differentiation via plasminogen activator inhibitor 1. Int J Mol Sci.

[26] Yu M., Wang L., Ba P. (2017). Osteoblast Progenitors Enhance Osteogenic Differentiation of Periodontal Ligament Stem Cells. J Periodontol.

[27] Montoya G., Arenas J., Romo E. (2014). Human recombinant cementum attachment protein (hrPTPLa/CAP) promotes hydroxyapatite crystal formation in vitro and bone healing in vivo. Bone.

[28] Wu D., Ikezawa K., Parker T., Saito M., Narayanan AS. (1996). Characterization of a collagenous cementum-derived attachment protein. J Bone Miner Res.

[29] Bar-Kana I., Savion N., Narayanan A.S., Pitaru S. (1998). Cementum attachment protein manifestation is restricted to the mineralized tissue forming cells of the periodontium. Eur J Oral Sci.

[30] Ureiro-Cueto G., Rodil S.E., Santana-Vázquez M., Hoz-Rodriguez L., Arzate H., Montoya-Ayala G. (2024). Characterization of aTiO2 surfaces functionalized with CAP-p15 peptide. J Biomed Mater Res A.

[31] Han P., Ivanovski S., Crawford R., Xiao Y. (2015). Activation of the canonical Wnt signaling pathway induces cementum regeneration. J Bone Miner Res.

[32] Pitaru S., Pritzki A., Bar-Kana I., Grosskopf A., Savion N., Narayanan AS. (2002). Bone morphogenetic protein 2 induces the expression of cementum attachment protein in human periodontal ligament clones. Connect Tissue Res.

[33] Miron R.J., Sculean A., Cochran D.L. (2016). Twenty years of enamel matrix derivative: the past, the present and the future. J Clin Periodontol.

[34] Kémoun P., Laurencin-Dalicieux S., Rue J. (2007). Human dental follicle cells acquire cementoblast features under stimulation by BMP-2/-7 and enamel matrix derivatives (EMD) in vitro. Cell Tissue Res.

[35] Bosshardt D.D., Sculean A., Windisch P., Pjetursson B.E., Lang NP. (2005). Effects of enamel matrix proteins on tissue formation along the roots of human teeth. J Periodontal Res.

[36] Hong H.H., Chou T.A., Hong A. (2022). Calcitriol and enamel matrix derivative differentially regulated cemento-induction and mineralization in human periodontal ligament-derived cells. J Periodontol.

[37] Kunimatsu R., Yoshimi Y., Hirose N. (2017). The C-terminus of amelogenin enhances osteogenic differentiation of human cementoblast lineage cells. J Periodontal Res.

[38] Reddi AH. (2005). BMPs: from bone morphogenetic proteins to body morphogenetic proteins. Cytokine Growth Factor Rev.

[39] Açil Y., Yang F., Gulses A., Ayna M., Wiltfang J., Gierloff M. (2016). Isolation, characterization and investigation of differentiation potential of human periodontal ligament cells and dental follicle progenitor cells and their response to BMP-7 in vitro. Odontology.

[40] E L.L., Zhang R., Li C.J. (2021). Effects of rhBMP-2 on Bone Formation Capacity of Rat Dental Stem/Progenitor Cells from Dental Follicle and Alveolar Bone Marrow. Stem Cells Dev.

[41] Zhao M., Xiao G., Berry J.E., Franceschi R.T., Reddi A., Somerman MJ. (2002). Bone morphogenetic protein 2 induces dental follicle cells to differentiate toward a cementoblast/osteoblast phenotype. J Bone Miner Res.

[42] Popowics T., Foster B.L., Swanson E.C., Fong H., Somerman MJ. (2005). Defining the roots of cementum formation. Cells Tissues Organs.

[43] Ding T., Li J., Zhang X., Du L., Li Y., Li D. (2020). Super-assembled core/shell fibrous frameworks with dual growth factors for in situ cementum-ligament-bone complex regeneration. Biomater Sci.

[44] Wei L., Teng F., Deng L. (2019). Periodontal regeneration using bone morphogenetic protein 2 incorporated biomimetic calcium phosphate in conjunction with barrier membrane: a pre-clinical study in dogs. J Clin Periodontol.

[45] Lee S.Y., Auh Q.S., Kang S.K., Kim H.J., Lee J.W., Noh K. (2014). Combined effects of dentin sialoprotein and bone morphogenetic protein-2 on differentiation in human cementoblasts. Cell Tissue Res.

[46] Aryal A C S, Islam MS. (2024). Potential role of BMP7 in regenerative dentistry. Int Dent J.

[47] Cho H., Tarafder S., Fogge M., Kao K., Lee CH. (2016). Periodontal ligament stem/progenitor cells with protein-releasing scaffolds for cementum formation and integration on dentin surface. Connect Tissue Res.

[48] Bozic D., Grgurevic L., Erjavec I. (2012). The proteome and gene expression profile of cementoblastic cells treated by bone morphogenetic protein-7 in vitro. J Clin Periodontol.

[49] Torii D., Tsutsui T.W., Watanabe N., Konishi K. (2016). Bone morphogenetic protein 7 induces cementogenic differentiation of human periodontal ligament-derived mesenchymal stem cells. Odontology.

[50] Cao Z., Zhang H., Zhou X. (2012). Genetic evidence for the vital function of Osterix in cementogenesis. J Bone Miner Res.

[51] Koba T., Watanabe K., Goda S. (2021). The effect of transforming growth factor beta 1 on the mineralization of human cementoblasts. J Endod.

[52] Chen J., Chen G., Yan Z. (2014). TGF-β1 and FGF2 stimulate the epithelial-mesenchymal transition of HERS cells through a MEK-dependent mechanism. J Cell Physiol.

[53] Itaya S., Oka K., Ogata K. (2017). Hertwig's epithelial root sheath cells contribute to formation of periodontal ligament through epithelial-mesenchymal transition by TGF-β. Biomed Res.

[54] Ripamonti U., Parak R., Klar R.M., Dickens C., Dix-Peek T., Duarte R. (2017). Cementogenesis and osteogenesis in periodontal tissue regeneration by recombinant human transforming growth factor-β3 : a pilot study in Papio ursinus. J Clin Periodontol.

[55] Farimani Z., Shamshiri A.R., Asl Roosta H., Akbari S., Bohlouli M. (2021). Regenerative benefits of using growth factors in treatment of periodontal defects: a systematic review and meta-analysis with trial sequential analysis on preclinical studies. J Tissue Eng Regen Med.

[56] Du W., Du W., Yu H. (2018). The role of fibroblast growth factors in tooth development and incisor renewal. Stem Cells Int.

[57] Bizenjima T., Seshima F., Ishizuka Y., Takeuchi T., Kinumatsu T., Saito A. (2015). Fibroblast growth factor-2 promotes healing of surgically created periodontal defects in rats with early, streptozotocin-induced diabetes via increasing cell proliferation and regulating angiogenesis. J Clin Periodontol.

[58] Cha J.K., Sun Y.K., Lee J.S., Choi S.H., Jung UW. (2017). Root coverage using porcine collagen matrix with fibroblast growth factor-2: a pilot study in dogs. J Clin Periodontol.

[59] Kitamura M., Akamatsu M., Machigashira M. (2011). FGF-2 stimulates periodontal regeneration: results of a multi-center randomized clinical trial. J Dent Res.

[60] Yu S.J., Lee J.S., Jung U.W., Park J.C., Kim B.O., Choi SH. (2015). Effect of fibroblast growth factor on injured periodontal ligament and cementum after tooth replantation in dogs. J Periodontal Implant Sci.

[61] Moore Y.R., Dickinson D.P., Wikesjö UM. (2010). Growth/differentiation factor-5: a candidate therapeutic agent for periodontal regeneration? A review of pre-clinical data. J Clin Periodontol.

[62] Lee J., Wikesjö UM. (2014). Growth/differentiation factor-5: pre-clinical and clinical evaluations of periodontal regeneration and alveolar augmentation–review. J Clin Periodontol.

[63] Yin X., Li Y., Li J. (2016). Generation and periodontal differentiation of human gingival fibroblasts-derived integration-free induced pluripotent stem cells. Biochem Biophys Res Commun.

[64] Lynch S.E., Williams R.C., Polson A.M. (1989). A combination of platelet-derived and insulin-like growth factors enhances periodontal regeneration. J Clin Periodontol.

[65] Zhang Y., Miron R.J., Li S., Shi B., Sculean A., Cheng X. (2015). Novel MesoPorous BioGlass/silk scaffold containing adPDGF-B and adBMP7 for the repair of periodontal defects in beagle dogs. J Clin Periodontol.

[66] Weng D., Stapf L., Kern M., Kohal RJ. (2021). Platelet-derived growth factor-modulated guided tissue regeneration with a bioresorbable membrane in class iii furcation defects: a histometric study in the monkey. Materials (Basel).

[67] Li T., Wang H., Jiang Y. (2023). LITTIP/Lgr6/HnRNPK complex regulates cementogenesis via Wnt signaling. Int J Oral Sci.

[68] Xu Y., Lv C., Zhang J. (2019). Intermittent parathyroid hormone promotes cementogenesis in a PKA- and ERK1/2-dependent manner. J Periodontol.

[69] Li X., Tian B.M., Deng D.K. (2022). LncRNA GACAT2 binds with protein PKM1/2 to regulate cell mitochondrial function and cementogenesis in an inflammatory environment. Bone Res.

[70] Hao Y., Wang Y., Du M. (2022). Effects of long noncoding RNA H19 on cementoblast differentiation, mineralisation, and proliferation. Acta Odontol Scand.

[71] Li X., Tian B.M., Yin Y. (2024). Melatonin ameliorates inflammation-induced mitochondrial dysfunction and cementoblastic differentiation in cells by regulating the METTL3/LINC01444/HSPD1 axis. Fundam Res.

[72] Liu G., Sun Q., Wu X. (2023). Clock genes are expressed in cementum and regulate the proliferation and mineralization of cementoblasts. In Vitro Cell Dev Biol Anim.

[73] Liu S., Zhou Y., Chen Y. (2022). Bmal1 promotes cementoblast differentiation and cementum mineralization via Wnt/β-catenin signaling. Acta Histochem.

[74] Fu L., Wang M., Zhu G. (2022). Corrigendum to REV-ERBs negatively regulate mineralization of the cementoblasts. Biochem Biophys Res Commun.

[75] Zhao Y., Huang Y., Liu H. (2022). Macrophages with different polarization phenotypes influence cementoblast mineralization through exosomes. Stem Cells Int.

[76] Liu J., Chen B., Bao J., Zhang Y., Lei L., Yan F. (2019). Macrophage polarization in periodontal ligament stem cells enhanced periodontal regeneration. Stem Cell Res Ther.

[77] Li X., He X.T., Kong D.Q. (2019). M2 macrophages enhance the cementoblastic differentiation of periodontal ligament stem cells via the Akt and JNK pathways. Stem Cells.

[78] Zhou N., Li N., Liu J. (2019). Persistent Wnt/β-catenin signaling in mouse epithelium induces the ectopic Dspp expression in cheek mesenchyme. Organogenesis.

[79] Nottmeier C., Liao N., Simon A. (2021). Wnt1 promotes cementum and alveolar bone growth in a time-dependent manner. J Dent Res.

[80] Choi H., Ahn Y.H., Kim T.H. (2016). TGF-β signaling regulates cementum formation through osterix expression. Sci Rep.

[81] Mo L., Zhu J., Li M. (2024). Smads and AP-1 activation of TGF-β signaling upregulate transcription of Osteoprotegerin in Cementoblasts to inhibit osteoclastogenesis. FASEB J.

[82] Bae W.J., Auh Q.S., Lim H.C., Kim G.T., Kim H.S., Kim EC. (2016). Sonic hedgehog promotes cementoblastic differentiation via activating the BMP pathways. Calcif Tissue Int.

[83] Morrison DK. (2012). MAP kinase pathways. Cold Spring Harb Perspect Biol.

[84] Ma L., Wang X., Liu H. (2019). CXXC5 mediates *P. gingivalis*-suppressed cementoblast functions partially via MAPK signaling network. Int J Biol Sci.

[85] Ma L., Liu H., Wang X. (2021). CXXC5 orchestrates Stat3/Erk/Akt signaling networks to modulate *P. gingivalis*-elicited autophagy in cementoblasts. Biochim Biophys Acta Mol Cell Res.

[86] Yoshimi Y., Kunimatsu R., Hirose N. (2016). Effects of C-terminal amelogenin peptide on proliferation of human cementoblast lineage cells. J Periodontol.

[87] Zhu J., Wang Y., Cao Z. (2020). Irisin promotes cementoblast differentiation via p38 MAPK pathway. Oral Dis.

[88] Yang B., Sun H., Song F., Wu Y., Wang J. (2018). Yes-associated protein 1 promotes the differentiation and mineralization of cementoblast. J Cell Physiol.

[89] Zhao Z., Dai J., Yin C. (2019). Transcription factor 7-like 2-associated signaling mechanism in regulating cementum generation by the NF-κB pathway. J Cell Physiol.

[90] Wang Y.L., He H., Liu Z.J. (2015). Effects of TNF-α on Cementoblast Differentiation, Mineralization, and Apoptosis. J Dent Res.

[91] Liao H.Q., Liu H., Sun H.L. (2019). MiR-361-3p/Nfat5 signaling axis controls cementoblast differentiation. J Dent Res.

[92] Li T., Wang H., Jiang Y. (2022). Canonical Wnt/β-catenin signaling has positive effects on osteogenesis, but can have negative effects on cementogenesis. J Periodontol.

[93] Cao Z., Liu R., Zhang H. (2015). Osterix controls cementoblast differentiation through downregulation of Wnt-signaling via enhancing DKK1 expression. Int J Biol Sci.

[94] Sculean A., Nikolidakis D., Nikou G., Ivanovic A., Chapple I.L., Stavropoulos A. (2015). Biomaterials for promoting periodontal regeneration in human intrabony defects: a systematic review. Periodontol 2000.

[95] Annunziata M., Piccirillo A., Perillo F., Cecoro G., Nastri L., Guida L. (2019). Enamel matrix derivative and autogenous bone graft for periodontal regeneration of intrabony defects in humans: a systematic review and meta-analysis. Materials (Basel).

[96] Keskiner I., Alkan A., Acikgoz G., Arpak N., Kaplan S., Arslan H. (2014). Platelet-rich plasma and autogenous bone graft combined with guided tissue regeneration in periodontal fenestration defects in dogs. Int J Periodontics Restorative Dent.

[97] Bottino M.C., Thomas V., Schmidt G. (2012). Recent advances in the development of GTR/GBR membranes for periodontal regeneration–a materials perspective. Dent Mater.

[98] Vahdatinia F., Hooshyarfard A., Jamshidi S. (2023). 3D-printed soft membrane for periodontal guided tissue regeneration. Materials (Basel).

[99] Abdo V.L., Suarez L.J., de Paula L.G. (2023). Underestimated microbial infection of resorbable membranes on guided regeneration. Colloids Surf B Biointerfaces.

[100] Liang Y., Luan X., Liu X. (2020). Recent advances in periodontal regeneration: a biomaterial perspective. Bioact Mater.

[101] Cicciù M. (2020). Growth factor applied to oral and regenerative surgery. Int J Mol Sci.

[102] Swanson W.B., Yao Y., Mishina Y. (2022). Novel approaches for periodontal tissue engineering. Genesis.

[103] França-Grohmann I.L., Sangiorgio J.P.M., Bueno M.R. (2020). Treatment of dehiscence-type defects with collagen matrix and/or enamel matrix derivative: Histomorphometric study in minipigs. J Periodontol.

[104] Shirakata Y., Miron R.J., Nakamura T. (2017). Effects of EMD liquid (Osteogain) on periodontal healing in class III furcation defects in monkeys. J Clin Periodontol.

[105] Cochran D.L., Jones A., Heijl L., Mellonig J.T., Schoolfield J., King GN. (2003). Periodontal regeneration with a combination of enamel matrix proteins and autogenous bone grafting. J Periodontol.

[106] Wang Z., Feng Z., Wu G., Bai S., Dong Y., Zhao Y. (2016). In vitro studies on human periodontal ligament stem cell sheets enhanced by enamel matrix derivative. Colloids Surf B Biointerfaces.

[107] Mijiritsky E., Assaf H.D., Peleg O., Shacham M., Cerroni L., Mangani L. (2021). Use of PRP, PRF and CGF in periodontal regeneration and facial rejuvenation-a narrative review. Biology (Basel).

[108] Masuki H., Okudera T., Watanebe T. (2016). Growth factor and pro-inflammatory cytokine contents in platelet-rich plasma (PRP), plasma rich in growth factors (PRGF), advanced platelet-rich fibrin (A-PRF), and concentrated growth factors (CGF). Int J Implant Dent.

[109] Nagata M.J., de Campos N., Messora M.R., Santinoni C.S., Bomfim S.R., Fucini S.E. (2014). Platelet-rich plasma derived from bone marrow aspirate promotes new cementum formation. J Periodontol.

[110] Rezaei M., Jamshidi S., Saffarpour A. (2019). Transplantation of bone marrow-derived mesenchymal stem cells, platelet-rich plasma, and fibrin glue for periodontal regeneration. Int J Periodontics Restorative Dent.

[111] Yang J.M., Yang K.I., Lee K.H. (2018). Effects of platelet-rich plasma on tooth replantation in dogs: a histologic and histomorphometric analysis. J Periodontal Implant Sci.

[112] Shirakata Y., Sena K., Nakamura T. (2021). Histological evaluation of gingival and intrabony periodontal defects treated with platelet-rich fibrin using different protocols: a canine study. Oral Health Prev Dent.

[113] Gollapudi M., Bajaj P., Oza RR. (2022). Injectable platelet-rich fibrin - a revolution in periodontal regeneration. Cureus.

[114] Miron R.J., Moraschini V., Fujioka-Kobayashi M. (2021). Use of platelet-rich fibrin for the treatment of periodontal intrabony defects: a systematic review and meta-analysis. Clin Oral Investig.

[115] Pullishery F., Hussein Alattas M., Roshdy Abdelrasoul M., Fouad Hassan A., Abdelhamid Ahmed Derbala D., Hashir S. (2024). Effectiveness of i-PRF in periodontal regeneration - a systematic review and meta-analysis. Saudi Dent J.

[116] Khijmatgar S., Panda S., Das M., Arbildo-Vega H., Del Fabbro M. (2021). Recombinant factors for periodontal intrabony defects: a systematic review and network meta-analysis of preclinical studies. J Tissue Eng Regen Med.

[117] Khehra A., Shiba T., Chen C.Y., Kim DM. (2024). Latest update on the use of recombinant growth factors for periodontal regeneration: existing evidence and clinical applications. Ther Adv Chronic Dis.

[118] Liu J., Ruan J., Weir M.D., Ren K., Schneider A., Wang P. (2019). Periodontal bone-ligament-cementum regeneration via scaffolds and stem cells. Cells.

[119] Chauca-Bajaña L., Velasquez-Ron B., Tomás-Carmona I., Camacho-Alonso F., Pérez-Jardón A., Pérez-Sayáns M. (2023). Regeneration of periodontal bone defects with mesenchymal stem cells in animal models. Systematic review and meta-analysis. Odontology.

[120] Zhang J., Ding H., Liu X., Sheng Y., Liu X., Jiang C. (2019). Dental follicle stem cells: tissue engineering and immunomodulation. Stem Cells Dev.

[121] Liu J., Zhao Z., Ruan J. (2020). Stem cells in the periodontal ligament differentiated into osteogenic, fibrogenic and cementogenic lineages for the regeneration of the periodontal complex. J Dent.

[122] Roato I., Masante B., Putame G., Massai D., Mussano F. (2022). Challenges of periodontal tissue engineering: increasing biomimicry through 3d printing and controlled dynamic environment. Nanomaterials (Basel).

[123] Li Q., Yang G., Li J. (2020). Stem cell therapies for periodontal tissue regeneration: a network meta-analysis of preclinical studies. Stem Cell Res Ther.

[124] Gaur S., Agnihotri R. (2021). Application of adipose tissue stem cells in regenerative dentistry: a systematic review. J Int Soc Prev Community Dent.

[125] Shi Z., Jia L., Zhang Q. (2022). An altered oral microbiota induced by injections of superparamagnetic iron oxide nanoparticle-labeled periodontal ligament stem cells helps periodontal bone regeneration in rats. Bioeng Transl Med.

[126] Li G., Han N., Zhang X. (2018). Local injection of allogeneic stem cells from apical papilla enhanced periodontal tissue regeneration in minipig model of periodontitis. Biomed Res Int.

[127] Owaki T., Shimizu T., Yamato M., Okano T. (2014). Cell sheet engineering for regenerative medicine: current challenges and strategies. Biotechnol J.

[128] Iwata T., Yamato M., Tsuchioka H. (2009). Periodontal regeneration with multi-layered periodontal ligament-derived cell sheets in a canine model. Biomaterials.

[129] Tavelli L., McGuire M.K., Zucchelli G. (2020). Extracellular matrix-based scaffolding technologies for periodontal and peri-implant soft tissue regeneration. J Periodontol.

[130] Samiei M., Alipour M., Khezri K. (2022). Application of collagen and mesenchymal stem cells in regenerative dentistry. Curr Stem Cell Res Ther.

[131] Yang T., Li Y., Hong Y. (2020). The construction of biomimetic cementum through a combination of bioskiving and fluorine-containing biomineralization. Front Bioeng Biotechnol.

[132] Shirakata Y., Imafuji T., Nakamura T. (2022). Cross-linked hyaluronic acid gel with or without a collagen matrix in the treatment of class III furcation defects: a histologic and histomorphometric study in dogs. J Clin Periodontol.

[133] Duruel T., Çakmak A.S., Akman A., Nohutcu R.M., Gümüşderelioğlu M. (2017). Sequential IGF-1 and BMP-6 releasing chitosan/alginate/PLGA hybrid scaffolds for periodontal regeneration. Int J Biol Macromol.

[134] Antunovic F., Tolosa F., Klein C., Ocaranza R. (2023). Polycaprolactone-based scaffolds for guided tissue regeneration in periodontal therapy: a systematic review. J Appl Biomater Funct Mater.

[135] Shah A.T., Zahid S., Ikram F. (2019). Tri-layered functionally graded membrane for potential application in periodontal regeneration. Mater Sci Eng C Mater Biol Appl.

[136] Sowmya S., Mony U., Jayachandran P. (2017). Tri-layered nanocomposite hydrogel scaffold for the concurrent regeneration of cementum, periodontal ligament, and alveolar bone. Adv Healthc Mater.

[137] Mao L., Liu J., Zhao J. (2015). Effect of micro-nano-hybrid structured hydroxyapatite bioceramics on osteogenic and cementogenic differentiation of human periodontal ligament stem cell via Wnt signaling pathway. Int J Nanomedicine.

[138] Zhou Y., Wu C., Xiao Y. (2014). Silicate-based bioceramics for periodontal regeneration. J Mater Chem B.

[139] Fakheran O., Birang R., Schmidlin P.R., Razavi S.M., Behfarnia P. (2019). Retro MTA and tricalcium phosphate/retro MTA for guided tissue regeneration of periodontal dehiscence defects in a dog model: a pilot study. Biomater Res.

[140] Liu J., Dai Q., Weir M.D. (2020). Biocompatible nanocomposite enhanced osteogenic and cementogenic differentiation of periodontal ligament stem cells in vitro for periodontal regeneration. Materials (Basel).

[141] Lee C.H., Hajibandeh J., Suzuki T., Fan A., Shang P., Mao J.J. (2014). Three-dimensional printed multiphase scaffolds for regeneration of periodontium complex. Tissue Eng Part A.

[142] Vaquette C., Fan W., Xiao Y., Hamlet S., Hutmacher D.W., Ivanovski S. (2012). A biphasic scaffold design combined with cell sheet technology for simultaneous regeneration of alveolar bone/periodontal ligament complex. Biomaterials.

[143] Ivanovski S., Vaquette C., Gronthos S., Hutmacher D.W., Bartold PM. (2014). Multiphasic scaffolds for periodontal tissue engineering. J Dent Res.

[144] Yu M., Luo D., Qiao J. (2021). A hierarchical bilayer architecture for complex tissue regeneration. Bioact Mater.

[145] Mei N., Wu Y., Chen B. (2022). 3D-printed mesoporous bioactive glass/GelMA biomimetic scaffolds for osteogenic/cementogenic differentiation of periodontal ligament cells. Front Bioeng Biotechnol.

[146] Ma Y., Yang X., Chen Y. (2023). Biomimetic peridontium patches for functional periodontal regeneration. Adv Healthc Mater.

[147] Ono T., Tomokiyo A., Ipposhi K. (2021). Generation of biohybrid implants using a multipotent human periodontal ligament cell line and bioactive core materials. J Cell Physiol.

[148] Wang T., Zhou Y., Zhang W. (2024). Exosomes and exosome composite scaffolds in periodontal tissue engineering. Front Bioeng Biotechnol.

[149] Deng D., Li X., Zhang J.J. (2023). Biotin-Avidin system-based delivery enhances the therapeutic performance of MSC-derived exosomes. ACS Nano.

[150] Huang X., Deng Y., Xiao J., Wang H., Yang Q., Cao Z. (2023). Genetically engineered M2-like macrophage-derived exosomes for *P. gingivalis*-suppressed cementum regeneration: From mechanism to therapy. Bioact Mater.

[151] Cui Y., Hong S., Xia Y. (2023). Melatonin engineering M2 macrophage-derived exosomes mediate endoplasmic reticulum stress and immune reprogramming for periodontitis therapy. Adv Sci (Weinh).

[152] Sun J., Wang Z., Liu P. (2022). Exosomes derived from human gingival mesenchymal stem cells attenuate the inflammatory response in periodontal ligament stem cells. Front Chem.

[153] Li S., Guan X., Yu W., Zhao Z., Sun Y., Bai Y. (2024). Effect of human periodontal ligament stem cell-derived exosomes on cementoblast activity. Oral Dis.

[154] Lv Z., Fu K., Zhang Q. (2023). Advances of exosomes-based applications in diagnostic biomarkers for dental disease and dental regeneration. Colloids Surf B Biointerfaces.

[155] Schmidt AH. (2021). Autologous bone graft: is it still the gold standard?. Injury.

[156] Galarraga-Vinueza M.E., Barootchi S., Nevins M.L., Nevins M., Miron R.J., Tavelli L. (2024). Twenty-five years of recombinant human growth factors rhPDGF-BB and rhBMP-2 in oral hard and soft tissue regeneration. Periodontol 2000.

[157] Mooney D.J., Vandenburgh H. (2008). Cell delivery mechanisms for tissue repair. Cell Stem Cell.

[158] Iwata T., Washio K., Yoshida T. (2015). Cell sheet engineering and its application for periodontal regeneration. J Tissue Eng Regen Med.

[159] Thalakiriyawa D.S., Dissanayaka WL. (2024). Advances in regenerative dentistry approaches: an update. Int Dent J.

[160] Liu Y., Graves D.T., Wang S. (2023). Development and clinical application of human mesenchymal stem cell drugs. Sci Bull (Beijing).

[161] Sone H., Kajiya M., Takeda K. (2022). Clumps of mesenchymal stem cells/extracellular matrix complexes directly reconstruct the functional periodontal tissue in a rat periodontal defect model. J Tissue Eng Regen Med.

[162] Rambhia K.J., Sun H., Feng K. (2024). Nanofibrous 3D scaffolds capable of individually controlled BMP and FGF release for the regulation of bone regeneration. Acta Biomater.

[163] Parisi L., Mavrogonatou E., Sculean A., Kletsas D., Degen M. (2024). Reviewing the benefits and clinical outcomes of oral fibroblasts over mesenchymal stem cells for repairing periodontal defects during or after orthodontic tooth movement. Periodontol 2000.

[164] Zhou L.L., Liu W., Wu Y.M., Sun W.L., Dörfer C.E., Fawzy El-Sayed K.M. (2020). Oral mesenchymal stem/progenitor cells: the immunomodulatory masters. Stem Cells Int.

[165] Zong C., Bronckaers A., Willems G., He H., Cadenas de Llano-Pérula M. (2023). Nanomaterials for periodontal tissue regeneration: progress, challenges and future perspectives. J Funct Biomater.

